# Patterns of complementary and alternative medicine and health care utilization among long-term opioid users with chronic non-cancer pain: a repeated cross-sectional study

**DOI:** 10.1186/s12906-026-05498-w

**Published:** 2026-08-03

**Authors:** Hannah Ahrensberg, Nanna Gram Ahlmark, Susan Andersen, Geana Paula Kurita, Per Føge Jensen, Ola Ekholm

**Affiliations:** 1 National Institute of Public Health, University of Southern Denmark, Studiestraede 6, Copenhagen, 1455 Denmark; 2 Department of Clinical Medicine, Faculty of Health and Medical Sciences, University of Copenhagen, Blegdamsvej 3, Copenhagen, 2200 Denmark; 3 Pain and Palliative Care Research Group, Multidisciplinary Pain Center, Department of Anaesthesiology, Pain and Respiratory Support, Copenhagen University Hospital – Rigshospitalet, Blegdamsvej 9, dept 7612, Copenhagen, 2100 Denmark; 4 St. Luke’s Academy of Palliative Medicine, St. Luke’s Foundation, Bernstorffsvej 20, Hellerup, 2900 Denmark

**Keywords:** Opioid use, Complementary and alternative medicine, Health care services, Chronic pain management, Cross-sectional study

## Abstract

**Background:**

High opioid use among individuals with chronic non-cancer pain (CNCP) raises concerns about safe pain management, leading some patients to seek complementary and alternative medicine (CAM). However, treatment-seeking patterns, including how CAM and conventional care are combined, remain unclear among those receiving long-term opioid treatment. The aim was to investigate health-seeking behaviours among individuals with CNCP receiving long-term opioid treatment.

**Methods:**

Repeated cross-sectional data from the Danish Health and Morbidity Surveys (2000–2021) were linked to national administrative registers at the individual level. Descriptive statistics and multiple linear and logistic regression models were applied to analyze CAM use, general practitioner (GP) consultations, physiotherapy, and chiropractic care within the last 12 months in relation to CNCP and long-term opioid use.

**Results:**

Trends remained largely unchanged from 2000 to 2021, with only minor fluctuations. GP consultations increased with long-term opioid use and were further elevated among individuals with CNCP who also used CAM. Among individuals with CNCP, combined long-term opioid and CAM use was associated with an average of 20.1 additional GP visits (95% CI: 17.5–22.7) compared with individuals without CNCP and no CAM use. Physiotherapy and chiropractic visits were more strongly associated with CAM use than with long-term opioid use. For instance, CNCP individuals using CAM without long-term opioid use had 5.8 times higher odds (95% CI: 4.6–7.4) of at least one chiropractor visit compared with those without CNCP and no CAM use.

**Conclusion:**

These findings highlight distinct health care‑seeking patterns and indicate a substantial health care burden among CNCP individuals receiving long‑term opioid treatment.

**Supplementary Information:**

The online version contains supplementary material available at https://doi.org/10.1186/s12906-026-05498-w.

## Background

Chronic pain represents a substantial public health burden, impacting physical, mental, and social well-being and generating considerable societal costs [3, 29]. A large proportion of the global population experiences chronic pain [3], and in Denmark, the prevalence of chronic non-cancer pain (CNCP) increased from 19.5% in 2000 to 28.9% in 2023 [5]. Although both pharmacological and non-pharmacological treatments are available, many patients continue to experience insufficient pain relief [37], highlighting persistent challenges in pain management and the need to better understand real-world treatment strategies.

As a result, many may turn to complementary and alternative medicine (CAM) as part of their pain management strategy. Recent evidence indicates increasing CAM use, with approaches such as massage and other manipulative therapies, acupuncture, reflexology, and craniosacral therapy being more frequently reported among individuals with CNCP compared to those without CNCP [1].

Rising CNCP prevalence has also intensified concerns regarding opioid use as a treatment approach [6]. Opioids are primarily indicated for acute pain, cancer pain, and for specific clinical contexts where alternatives are limited [25, 26]. However, they are frequently used for CNCP within conventional health care systems, despite well-known risks associated with long-term use, including dependence, tolerance, and adverse health outcomes [2]. Denmark ranks among the countries with the highest opioid consumption worldwide (measured in morphine milligram equivalents) at the population level [14], and a substantial proportion (58.2%) of opioid users without cancer in Denmark have CNCP [10].

While previous studies have examined trends in opioid use [11, 14, 20] and adoption of CAM in relation to health care utilization among individuals with and without CNCP [1], no studies have examined how opioid use relates to CAM utilization or how these treatment modalities co-occur within broader treatment patterns of health care use. Understanding the relationships between long-term opioid use, CAM use, and conventional health care utilization is critical, as it can reveal limitations in pain management approaches and indicate where current strategies may fall short. Moreover, examining trends over time provides insights into evolving treatment preferences and informs clinical practice and policy efforts aimed at improving pain management.

Therefore, this study aims to examine trends and patterns of long-term opioid use in relation to CAM use and conventional health care utilization among individuals with CNCP, and to compare these patterns with those observed in individuals without CNCP.

## Methods

### Study design and participants

**Fig. 1:**
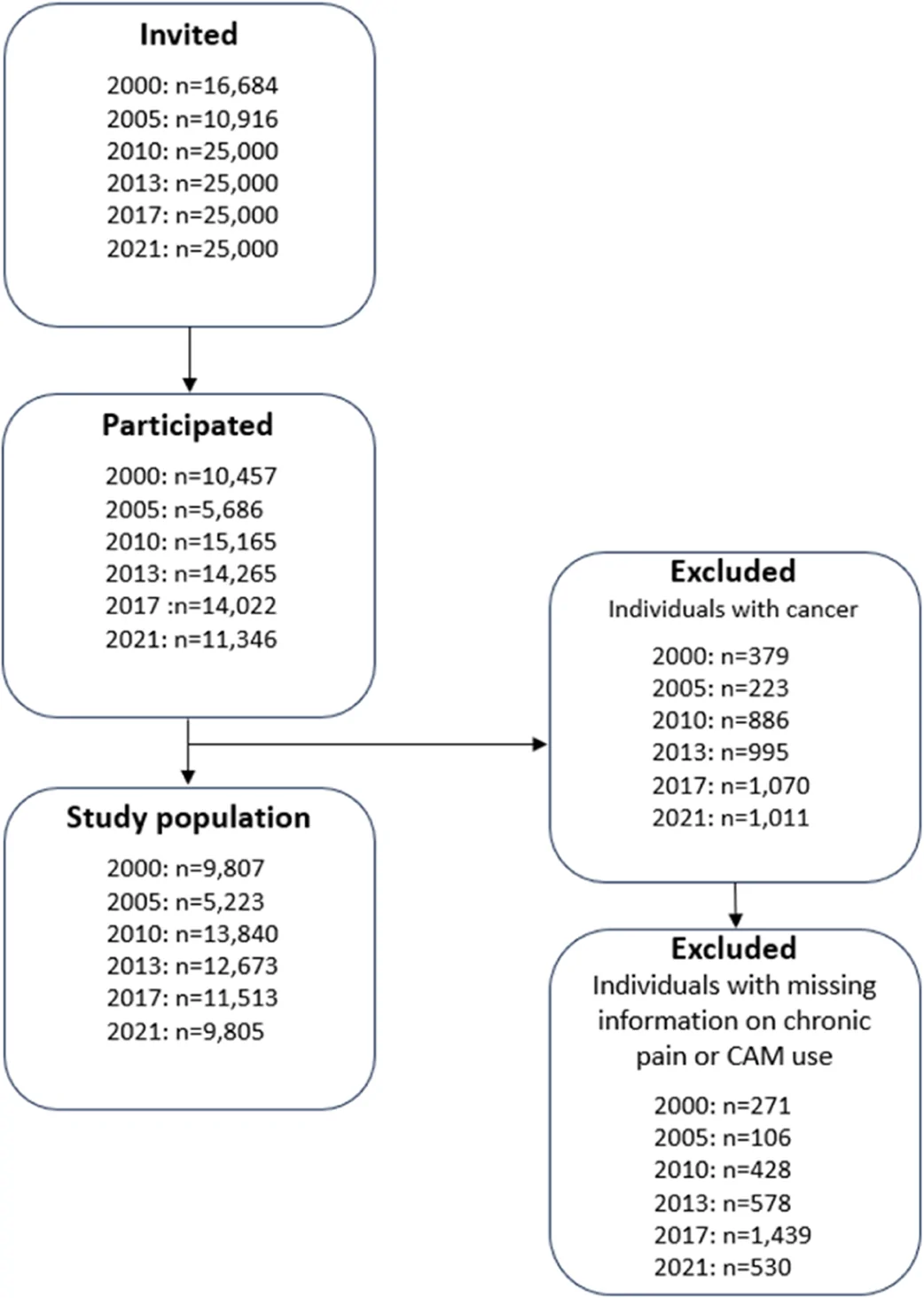
Flowchart of study participants in 2000, 2005, 2010, 2013, 2017, and 2021 in the Danish health and morbidity surveys

This study employed repeated cross-sectional data from the Danish Health and Morbidity Surveys (DHMS), conducted in 2000, 2005, 2010, 2013, 2017, and 2021, in conjunction with data from national administrative registers. DHMS is a well-established national survey designed for population health monitoring and has been described in detail elsewhere [12]. DHMS assesses health status, morbidity, and factors influencing health among the individuals aged 16 years or older in Denmark [12]. Samples were randomly selected from the Danish Civil Registration System [27], and the use of personal identification numbers assigned to all residents with permanent residency in Denmark enable precise linkage between survey responses and register-based data sources [35]. Data collection methods varied across survey waves (Supplementary Table S1). In 2000 and 2005, only Danish citizens were sampled (16,684 and 10,916 invited, respectively), and data were collected through face-to-face interviews and self-administered paper questionnaires. In 2010, 2013, 2017, and 2021, both Danish and non-Danish citizens were included in the sample frame, with 25,000 invited to participate in each survey wave. In these years, data were collected through self-administered questionnaires sent by postal mail and, from 2017 onwards, also via the electronic mail service (Digital Post). A total of 10,457 (response rate: 63%), 5,686 (52%) in 2005, 15,165 (61%) in 2010, 14,265 (57%) in 2013, 14,022 (56%) in 2017, and 11,346 (45%) in 2021 participated in the respective survey waves. Individuals with a history of cancer recorded in the Danish National Patient Registry [31] (ICD-10: C00-C97; ICD-8: 140–208) were excluded (2000: *n* = 379; 2005: *n* = 223; 2010: *n* = 886; 2013: *n* = 995; 2017: *n* = 1,070; 2021: *n* = 1,011). In addition, individuals with missing information on self-reported chronic pain or CAM use were excluded (2000: *n* = 271; 2005: *n* = 106; 2010: *n* = 428; 2013: *n* = 578; 2017: *n* = 1,439; 2021: *n* = 530). The final study population consisted of 9,807 in 2000, 5,223 in 2005, 13,840 in 2010, 12,673 in 2013, 11,513 in 2017, and 9,805 in 2021 (Fig. 1).

### Measures

#### Chronic non-cancer pain

Chronic pain was assessed using the question: “Do you have chronic/long-lasting pain lasting 6 months or more?” with response options “Yes” and “No”. This question has been widely used in previous studies [4, 6, 16, 33]. Although the International Association of the Study of Pain redefined chronic pain in 2019 as pain persisting or recurring for more than three months [36], the six-month threshold was retained in 2021 to ensure comparability with earlier survey waves in DHMS. As described previously, individuals with a cancer (current or previous) diagnosis were excluded, as the mechanisms and management strategies for cancer-related pain differ.

#### Prescription of opioids

Data on dispensed opioids were retrieved from the Danish National Prescription Registry, which systematically records all prescription medications dispensed at pharmacies throughout Denmark [28]. Opioid prescriptions dispensed within the 12 months preceding data collection were identified using the Anatomical Therapeutic Chemical (ATC) classification system, specifically the codes: N02A (including all subcategories), N02BE51, N02BA51 and R05BA04. Long-term opioid use was defined as the dispensation of three or more opioid prescriptions within the specified 12-month period, a definition that has been used in several studies both in Denmark and in other countries [18, 23, 24].

#### Complementary and alternative medicine

CAM use was assessed with the question: “Have you ever received treatment from providers outside the conventional health care system and, for example, used any of the following providers or forms of treatment?”. Examples included massage and other manipulative therapies, acupuncture, and reflexology. A complete list of all specific types of CAMs used in each survey wave has been described elsewhere [21]. Respondents who affirmed one or more of the options were also asked whether they had used CAM within the past year. Only CAM use within the last 12 months was included in this study.

#### Health care utilization

Health care services, including consultations with general practitioners (GP), physiotherapists, and chiropractors, were obtained from the Danish National Health Service Register [30]. This register records all primary health care services provided in Denmark, including those from private providers. In Denmark, the health care system is universal, ensuring free and equal access to all residents, with funding through general taxation. This applies to GP consultations, while physiotherapy is subsidized when referred by a GP, and chiropractic care is not. Chiropractors were officially incorporated into the public health care system in Denmark in 1991, and since then, they have not been considered an alternative treatment option, as they are in some other countries. Accordingly, chiropractic care was analyzed separately from CAM in this study. Only health care services used in the 12 months preceding data collection were included in the present study. Additionally, the number of GP consultations was treated as a continuous variable, while visits to physiotherapists and chiropractors were categorized as either ‘Yes’ or ‘No’, since data showed that these therapies were less frequently used.

#### Sociodemographic variables and information on smoking status

Sex and age were retrieved from the Danish Civil Registration System, while cohabitation status was obtained by combining data on marital status from the Danish Civil Registration System and self-reported cohabitation. Highest completed educational level was obtained from the Danish Education Registers [13]. The categories of each variable can be found in Table 1. Furthermore, country of origin was classified based on information on the respondents’ birthplace, citizenship and parental birthplace. The information was obtained from the Danish Civil Registration System for all respondents in 2021. The respondents were categorized into three broad groups: Denmark; Western countries (e.g., all European Union member states, Norway, Iceland, UK, Canada, the United States, Australia and New Zealand); and non-Western countries. Self-reported smoking status was obtained for respondents in 2021 and categorized as current, former, and never smokers.

**Table 1:** Characteristics of respondents without a cancer history in each of the survey waves. Percentages

	2000	2005	2010	2013	2017	2021
Sex						
Men	48.1	47.0	46.5	45.7	45.1	43.8
Women	51.9	53.0	53.5	54.3	54.9	56.2
Age						
16–24 y.	13.0	9.0	11.8	12.9	11.4	10.6
25–44 y.	36.4	34.6	29.6	26.3	26.3	21.3
45–64 y.	35.6	38.6	38.3	37.8	37.7	36.4
65–79 y.	12.1	14.8	16.8	19.5	21.0	25.9
≥ 80 y.	2.8	3.0	3.4	3.0	3.6	5.9
Education						
Basic school	33.4	28.5	27.6	25.5	21.7	21.9
Upper secondary or vocational school	41.6	42.5	41.4	41.9	41.2	39.4
Higher education	20.0	26.7	28.5	30.4	34.6	37.3
No information	5.0	2.2	2.6	2.2	2.5	1.3
Cohabitation status						
Married	55.2	58.4	58.9	56.5	53.4	53.0
Cohabiting	15.9	15.3	12.4	13.0	16.1	14.8
Single (divorced, separated, widowed)	11.3	11.0	11.7	10.9	12.3	13.8
Single (unmarried)	17.6	15.3	16.8	19.6	18.2	18.4
Chronic non-cancer pain^1^						
Yes	18.8	20.4	25.6	26.3	27.9	25.5
No	81.2	79.6	74.4	73.7	72.1	74.5
Chronic non-cancer pain and long-term opioid user^1^						
Yes	1.8	1.9	2.5	2.8	3.0	2.4
No	98.2	98.1	97.5	97.2	97.0	97.6
Number of respondents	9,807	5,223	13,840	12,673	11,513	9,805

^1^Weighted for non-response

### Statistical analysis

Descriptive statistics were used to characterize the study population (percentages), examine trends in CAM use, physiotherapy, and chiropractic care (percentages), and examine treatment patterns for GP consultations, physiotherapy, and chiropractor visits by CNCP status, long-term opioid use, and CAM use (means and standard deviations). Associations between CNCP status with and without long-term opioid use and CAM use and health care utilization were then examined using multiple linear regression for GP consultations (β coefficients) and logistic regression for physiotherapy and chiropractic visits (odds ratios (ORs) with 95% confidence intervals (CI)). Analyses of CAM use and health care visits were based on utilization within the past 12 months, using data from the 2021 wave. All regression models were adjusted for potential confounders identified in prior literature, including sex, age, educational attainment, cohabitation status, country of origin, and smoking status [8, 15–17]. To enhance transparency, both adjusted and unadjusted models were presented, allowing for comparison of effect estimates before and after controlling for covariates. Participants with missing data on any variables required for a given analysis were excluded from that analysis.

Analyses involving education as a covariate were restricted to respondents aged 25 years or older, under the assumption that most individuals have completed their formal education by this age. To mitigate potential non-response bias, calibration weights were applied. These weights were computed by Statistics Denmark based on demographic and socioeconomic characteristics of all individuals invited to participate, including sex, age, highest completed education, employment status, income, marital status, country of origin, and health care utilization. All statistical analyses were conducted using SAS version 9.4.

## Results

Table 1 presents the characteristics of the study populations in each survey wave from 2000 to 2021 among individuals without a cancer history.

The proportion of individuals with CNCP increased from 18.8% in 2000 to 27.9% in 2017, before declining to 25.5% in 2021. Similarly, long-term opioid use among individuals with CNCP increased from 1.8% in 2000 to 3.0% in 2017, before declining to 2.4% in 2021. Across all survey waves, women, individuals aged 45–64 years, those with upper secondary or vocational education or higher, and married individuals were most likely to respond.

**Fig. 2:**
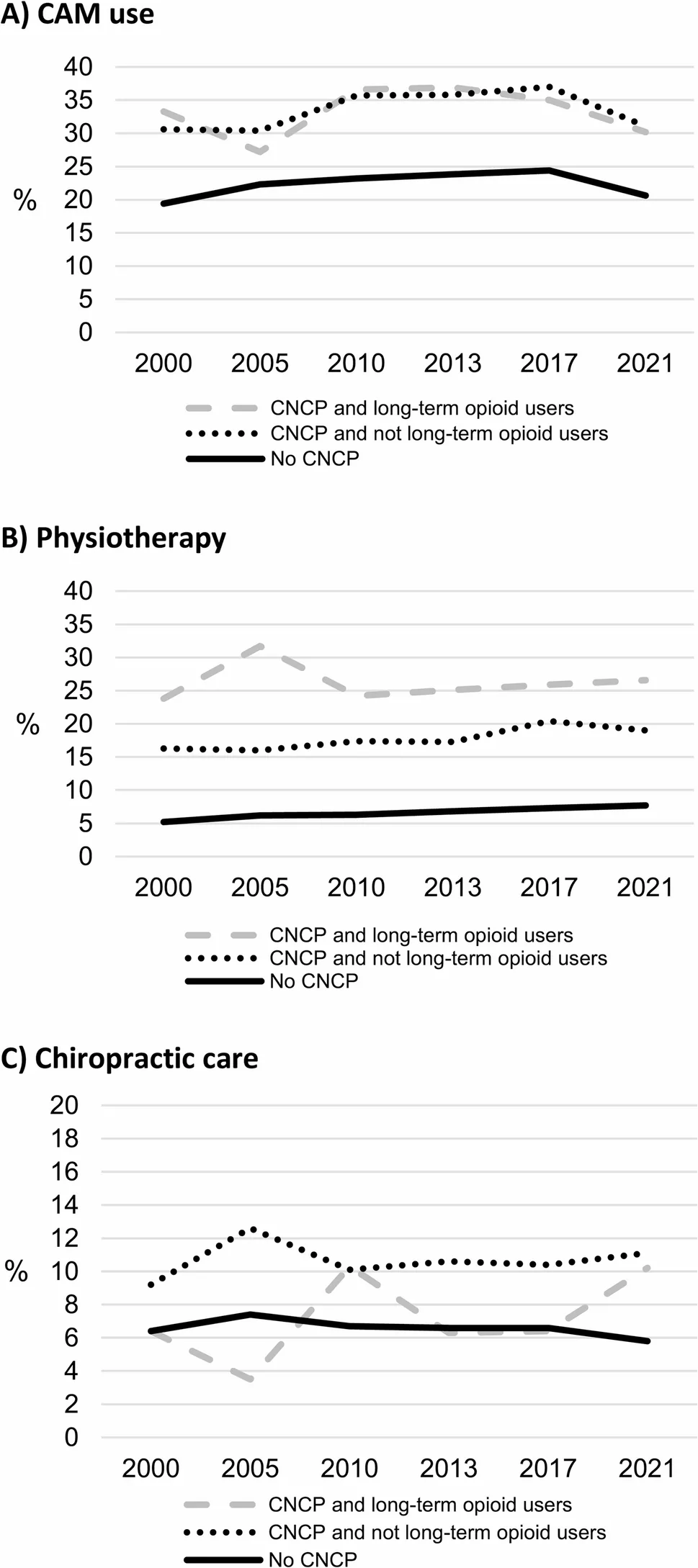
Trends in CAM use (**A**), physiotherapy (**B**), and chiropractic care (**C**) within the past 12 months by CNCP status and opioid use. Percentages

Figure 2 shows trends in CAM use (2A), physiotherapy (2B), and chiropractic care (2C) within the past 12 months by CNCP status and opioid use.

Overall, trends remain largely unchanged from 2000 to 2021, despite minor fluctuations across groups. CAM use increased modestly from 2000 to 2017 across CNCP and opioid use groups but declined again in 2021 (Fig. 2A). Physiotherapy use showed a small peak around 2005 among CNCP individuals with long-term opioid use before returning to levels comparable to those observed in 2000 (Fig. 2B). Chiropractic care demonstrated more variability over time among CNCP long-term opioid users, yet displayed an overall upward trend from 6.4% in 2000 to 10.2% in 2021 (Fig. 2C).

Across all survey waves, a higher proportion of individuals with CNCP used CAM (Fig. 2A) and physiotherapy (Fig. 2B) compared to those without CNCP. The largest differences are observed for physiotherapy: in 2021, 26.6% of individuals with CNCP and long-term opioid use reported physiotherapy use, compared with 19.0% of those with CNCP but no long-term opioid use, and 7.7% of individuals without CNCP (Fig. 2B). Differences in chiropractic care are less pronounced, although individuals with CNCP without long-term opioid use still tend to use chiropractic services more than both long-term opioid users with CNCP and individuals without CNCP (Fig. 2C). Exact percentages are provided in Supplementary Table S2. Age-adjusted percentages remain relatively stable across survey waves, apart from the 2000 and 2005 waves, where smaller sample sizes led to greater fluctuation (Supplementary Table S3).

Table 2 shows the mean number of GP consultations, visits to a physiotherapist, and a chiropractor, respectively, within the past 12 months by CNCP status, opioid use, and CAM use in 2021.

**Table 2:** Mean (SD) number of GP consultations, physiotherapist visits, and chiropractor visits, respectively, within the past 12 months by CNCP status, long-term opioid use, and CAM use within the past 12 months (2021)

	CAM use	No CAM use
GP consultations		
CNCP		
Long-term opioid users	29.3 (17.3)	27.4 (19.3)
Not long-term opioid users	15.4 (13.5)	14.3 (13.6)
No CNCP	10.7 (10.6)	8.4 (10.2)
Physiotherapist		
CNCP		
Long-term opioid users	6.6 (17.7)	4.0 (13.6)
Not long-term opioid users	4.5 (13.4)	2.2 (9.4)
No CNCP	1.3 (6.2)	0.6 (5.3)
Chiropractor		
CNCP		
Long-term opioid users	1.2 (3.1)	0.4 (2.0)
Not long-term opioid users	1.5 (4.2)	0.5 (2.0)
No CNCP	0.7 (2.5)	0.2 (1.3)

*CNCP* Chronic non-cancer pain, *CAM* Complementary and alternative medicine

Among individuals with CNCP, long-term opioid users have substantially higher GP service use than non-users, averaging 29.3 consultations for CAM users and 27.4 consultations for non-CAM users. In comparison, CNCP individuals without long-term opioid use have 15.4 and 14.3 consultations, respectively, while individuals without CNCP have an average of 10.7 and 8.4 consultations.

The mean number of physiotherapy visits is also higher among long-term opioid users (6.6 visits for CAM users and 4.0 for non-CAM users) than non-users (4.5 and 2.2, respectively). In contrast, differences in the mean number of chiropractic visits are smaller. Within each CNCP and opioid use group, CAM users generally have slightly higher mean number of visits to GP, physiotherapy, and chiropractic services than non-CAM users.

Table 3 shows associations between number of GP consultations, physiotherapist visits, and chiropractic visits by CNCP status, opioid use, and CAM use within the past 12 months in 2021, based on multiple linear and logistic regression models, respectively.

**Table 3:** Associations between number of GP consultations, physiotherapist visits, and chiropractor visits, respectively, and CNCP status, opioid use, and CAM use within the past 12 months (2021). β coefficients and odds ratios (OR), respectively, with 95% CI intervals

	Chronic non-cancer pain				No chronic non-cancer pain		*p*
	Long-term opioid use and CAM use	Long-term opioid use and no CAM use	No long-term opioid use and CAM use	No long-term opioid use and no CAM use	CAM use	No CAM use	
GP consultations (β coefficients)							
Model 1	21.0 (18.3–23.7)	19.0 (17.2–20.8)	7.1 (6.2-8.0)	5.9 (5.3–6.5)	2.3 (1.7-3.0)	0	*< 0.0001*
Model 2	20.0 (17.4–22.5)	16.2 (14.5–17.9)	6.4 (5.5–7.2)	4.8 (4.2–5.4)	2.4 (1.8-3.0)	0	*< 0.0001*
Model 3	20.1 (17.5–22.7)	15.5 (13.7–17.2)	6.0 (5.1–6.9)	4.5 (3.9–5.2)	2.5 (1.9–3.2)	0	*< 0.0001*
Physiotherapist (OR)							
Model 1	10.4 (6.4–17.0)	4.0 (2.7-6.0)	6.4 (5.2–7.7)	2.6 (2.2–3.1)	2.4 (2.0-2.9)	1	*< 0.0001*
Model 2	8.9 (5.4–14.6)	2.9 (1.9–4.3)	5.6 (4.6–6.9)	2.2 (1.9–2.7)	2.4 (2.0-2.9)	1	*< 0.0001*
Model 3	9.9 (5.9–16.6)	3.2 (2.1–4.8)	5.9 (4.8–7.3)	2.3 (1.9–2.8)	2.2 (1.8–2.6)	1	*< 0.0001*
Chiropractor (OR)							
Model 1	5.2 (2.8–9.7)	1.9 (1.0-3.4)	6.2 (4.9–7.7)	1.9 (1.5–2.3)	3.7 (3.0-4.9)	1	*< 0.0001*
Model 2	4.7 (2.5–8.8)	1.7 (0.9–3.1)	5.7 (4.6–7.2)	1.7 (1.4–2.2)	3.5 (2.9–4.3)	1	*< 0.0001*
Model 3	4.8 (2.5–9.1)	1.9 (1.0-3.6)	5.8 (4.6–7.4)	1.9 (1.5–2.4)	3.3 (2.6-4.0)	1	*< 0.0001*

*CNCP* Chronic non-cancer pain*, CAM* Complementary and alternative medicine

Model 1: Unadjusted

Model 2: Adjusted for sex and age

Model 3: Further adjusted for highest completed level of education, cohabitation status, country of origin, and smoking status (restricted to individuals aged 25 years or older)

Among CNCP individuals, long‑term opioid users who also used CAM had, on average, 20.1 more GP consultations (95% CI: 17.5–22.7) compared with individuals without CNCP and non-CAM users (linear regression, model 3). Overall, GP consultations increased progressively with CNCP, long-term opioid use, and CAM-use. In contrast, physiotherapist and chiropractor visits were more strongly associated with CAM use than with long-term opioid use. Among CAM-users, CNCP individuals with long-term opioid use have 9.9 times higher odds of physiotherapist visits (95% CI: 5.9–15.6), whereas CNCP individuals without long-term opioid use have 5.9 higher odds (95% CI: 4.8–7.3). Among non-CAM users, CNCP individuals with long-term opioid use have 3.2 times higher odds (95% CI: 2.1–4.8) of physiotherapist visits.

For chiropractic care, differences were less pronounced. Among CAM users, CNCP with long-term opioid use have similar odds of visiting a chiropractor as CNCP without long-term opioid use (OR 4.8 (95% CI: 2.5–9.1) vs. OR 5.8 (95% CI: 4.6–7.4)). Among non-CAM users, long-term opioid users have only 1.9 times higher odds (95% CI: 1.0-3.6) of visiting a chiropractor compared with individuals without CNCP and no long-term opioid use.

## Discussion

Our study examined trends and patterns of long-term opioid use in relation to CAM use and conventional health care utilization among individuals with and without CNCP.

From 2000 to 2021, the prevalence of CNCP and long-term opioid use increased, while the use of CAM, physiotherapy, and chiropractic care remained relatively stable despite minor fluctuations. This suggests that rising symptom burden and growing awareness have not translated into a marked shift toward greater uptake of non-pharmacological management strategies over the past two decades. It should be noted, however, that data from 2021 were collected during the COVID-19 pandemic, which may have temporarily affected access to and use of both CAM and conventional health care services. National register data also indicate a 12% reduction in prescription opioid use between 2018 and 2023, largely attributed to stricter prescribing practices for tramadol and efforts to limit the health-related consequences of opioid use [34]. Continued surveillance is, therefore, important to determine whether these regulatory changes lead to changes in care-seeking behaviour among individuals with CNCP.

Despite stable overall trends, pronounced differences in health care utilization were observed. GP consultations followed a graded pattern, increasing progressively with the presence of long-term opioid use among CNCP individuals and with CAM use. This pattern aligns with previous research showing that increased pain severity, longer pain duration, and opioid use are strong predictors of frequent GP contact [32]. Supporting previous findings that chronic pain patients using opioids report higher levels of depression, anxiety, and pain severity compared with non-users [9], our findings may suggest that long-term opioid use serves as an indicator of more severe or complex pain conditions accompanied by greater health care needs. In this context, high GP utilization among long-term opioid users likely reflects ongoing monitoring, prescription management, and broader health-related concerns.

Physiotherapy and chiropractic care were more strongly associated with CAM use than with opioid use alone. This suggests that individuals engaging in CAM may represent a subgroup with a broader, more proactive treatment-seeking profile, characterized by greater use of non-pharmacological and self-initiated strategies for managing chronic pain. Previous studies have similarly shown that practitioner-based CAM use often co-occurs with a wider range of health care service use [15, 22] and has been associated with lower inpatient costs [19], indicating that CAM may complement or partially substitute pharmacological pain management. Research also shows that individuals with chronic pain treated with prescription opioids often seek complementary or alternative treatment modalities alongside conventional care [7]. Similarly, CNCP individuals in our study with both long-term opioid use and CAM use had the highest odds of physiotherapist visits. In contrast, chiropractic care was most common among CNCP individuals using CAM but without long-term opioid use. This pattern may reflect that long-term opioid users may rely more on physiotherapy as part of ongoing management of chronic and persistent pain, whereas chiropractic care may represent a more direct response to acute or episodic musculoskeletal pain, for which opioids are less commonly indicated. It may also indicate that individuals without long-term opioid use are in earlier phases of their pain trajectory and may therefore be more likely to experiment with a broader range of treatment options, including chiropractic care. However, causal or temporal interpretations cannot be made due to the cross-sectional design of the study. Additionally, in Denmark, chiropractic care is primarily financed out-of-pocket, whereas physiotherapy is partly reimbursed when prescribed by a GP. Financial considerations may therefore influence patterns of use, potentially leading some patients to discontinue chiropractic care over time or not initiate at all. However, these differences are likely multifactorial and may also be influenced by treatment preferences, perceived effectiveness, symptom severity and characteristics, and referral patterns.

### Strengths and limitations

Strengths of this study include the use of repeated cross-sectional survey data linked with national registers at individual level, allowing for a detailed examination of trends in CAM use and health care utilization over a 21-year period. The large, population-based sample and linkage to register data provide robust and comprehensive coverage of health care contacts and prescriptions.

Limitations include the cross-sectional design, which prevents causal inferences, as well as declining response rates that could lead to selection bias. This was partly mitigated by weighting the data for non-response. Self-reported data were also used for both chronic pain and CAM use, which may introduce recall bias or variations in how respondents understand or classify chronic pain and specific CAM modalities. Nonetheless, given that chronic pain is inherently subjective and cannot be directly measured with objective instruments, self‑reports remain the most essential method for its assessment. Although long-term opioid use could be identified, information on dosing, adherence, and clinical indication was limited, which may have affected the interpretation of the observed patterns of health care-seeking behaviour, including CAM use and health care utilization. Prescription register data reflects dispensing rather than actual consumption but using a ≥ 3 prescription threshold strengthens our definition, as it likely captures individuals who have actually used opioids over time, considering the cost of obtaining the medication. This threshold, together with the inclusion of individuals of self-reported chronic pain, reduced the likelihood of substantial misclassification of opioid use in relation to acute or short-term pain. Nevertheless, some prescriptions may have been issued for non-musculoskeletal chronic pain conditions. Also, direct assessment of pain severity or duration was not possible; long-term opioid use may partially serve as a proxy for more severe or persistent pain, but finer-grained measures of pain characteristics could help clarify the drivers of GP and CAM use. Furthermore, residual confounding cannot be excluded. Although the analyses were adjusted a priori for key sociodemographic characteristics identified in previous literature, other factors such as mental health, other lifestyle factors than smoking status, and comorbidities were not included in the adjusted analyses. Some of these factors may represent true confounders, whereas others may lie on the causal pathway between CNCP, long-term opioid use, CAM use, and health care utilization.

Future research should explore why opioid users more frequently visit GPs, as the current study cannot disentangle whether this is driven by pain severity, co-occurring conditions, or other factors. Further examination of how pain characteristics, opioid use, and engagement with complementary therapies interact could also help clarify the balance between self-initiated and conventional guided care. In addition, it will be important to monitor national initiatives aimed at tightening opioid prescribing practices to understand whether these policy changes lead to a shift toward non-pharmacological treatments or alter long-term patterns of health care use among individuals with chronic pain.

## Conclusion

This study found that long-term opioid use among individuals with CNCP was consistently associated with higher GP consultations, whereas CAM use - regardless of long-term opioid status – was more strongly associated with increased visits to physiotherapists and chiropractors. These findings highlight distinct patterns of health care utilization tied to different CNCP management strategies. Understanding these associations can help inform strategies to optimize pain management and resource allocation, ensuring comprehensive and effective care for individuals with CNCP. Continued monitoring may reveal shifts in treatment patterns as opioid prescribing practices evolve.

## Supplementary Information

Supplementary Material 1.

## Data Availability

The datasets used and analyzed in the current study are not publicly available because they contain information that could compromise participants’ privacy. However, data may be made available from the corresponding author upon reasonable request.

## Abbreviations

CNCP: Chronic non-cancer pain
CAM: Complementary and alternative medicine
DHMS: Danish health and morbidity surveys
ICD-10: International classification of diseases, 10th revision
ATC: Anatomical therapeutic chemical classification system
GP: General practitioner
OR: Odds ratio
CI: Confidence interval
