# Supplementary material for: Patterns of complementary and alternative medicine and health care utilization among long-term opioid users with chronic non-cancer pain: a repeated cross-sectional study
Source: BMC Complement Med Ther. 2026 Aug 3;26:314. doi: 10.1186/s12906-026-05498-w (PMC13688784; doi:10.1186/s12906-026-05498-w)
Supplement: Supplementary file 1 [file 12906_2026_5498_MOESM1_ESM.pdf]

Table S1. Overview of the data collection methods and invited participants in the Danish Health and Morbidity Surveys conducted in 2000, 2005, 2010, 2013, 2017 and 2021.

	**2000**	**2005**	**2010**	**2013**	**2017**	**2021**
Face-to-face interviews	X	X				
Paper questionnaire	X	X	X	X	X	X
Web questionnaire			X	X	X	X
No. of invited	16,684	10,916	25,000	25,000	25,000	25,000

Table S1 reproduced without modifications from: Ahrensberg H, Ekholm O, Andersen S, Kurita GP, Jensen PF, Ahlmark NG. Trends in complementary and alternative medicine use and its association with healthcare services among individuals with chronic non-cancer pain: an analysis of repeated nationwide cross-sectional surveys linked to register data. The Journal of Pain 2025:105451, with permission.

Table S2. Trends in CAM use, physiotherapy and chiropractic care within the past 12 months by CNCP status and opioid use. Percentages.

	2000	2005	2010	2013	2017	2021
**CAM use**						
CNCP						
Long-term opioid users	33.3	27.2	36.6	36.9	35.0	30.2
Not long-term opioid users	30.6	30.4	35.7	35.8	37.0	31.1
No CNCP	19.4	22.3	23.2	23.8	24.4	20.6
**Physiotherapy**						
CNCP						
Long-term opioid users	23.8	31.7	24.2	25.1	25.9	26.6
Not long-term opioid users	16.3	16.0	17.4	17.3	20.4	19.0
No CNCP	5.2	6.2	6.3	6.8	7.3	7.7
**Chiropractic care**						
CNCP						
Long-term opioid users	6.4	3.5	10.3	6.3	6.4	10.2
Not long-term opioid users	9.2	12.6	10.1	10.6	10.4	11.1
No CNCP	6.4	7.4	6.7	6.6	6.6	5.8

CNCP: Chronic non-cancer pain

CAM: Complementary and alternative medicine

Table S3. Trends in CAM use, physiotherapy and chiropractic care within the past 12 months by CNCP status and opioid use. Age-adjusted percentages.

	2000	2005	2010	2013	2017	2021
**CAM use**						
CNCP						
Long-term opioid users	42.0	29.7	38.7	37.6	37.9	37.9
Not long-term opioid users	30.7	29.5	35.1	35.5	36.7	31.5
No CNCP	18.5	20.8	21.2	22.5	23.2	20.2
**Physiotherapy**						
CNCP						
Long-term opioid users	27.5	32.5	24.1	27.3	25.7	26.9
Not long-term opioid users	15.9	16.1	17.0	17.4	20.5	18.5
No CNCP	5.5	6.4	6.6	7.2	7.8	8.0
**Chiropractic care**						
CNCP						
Long-term opioid users	7.5	3.5	12.2	7.1	7.0	10.7
Not long-term opioid users	9.1	12.2	10.0	10.5	10.3	11.2
No CNCP	6.0	7.4	6.6	6.6	6.6	5.9

CNCP: Chronic non-cancer pain

CAM: Complementary and alternative medicine
